# Evidências Genéticas Reforçam o Papel Causal do Tabagismo na Parada Cardíaca

**DOI:** 10.36660/abc.20260238

**Published:** 2026-04-30

**Authors:** José S. L. Patané, Fernando R. Giugni

**Affiliations:** 1 Hospital das Clínicas da Faculdade de Medicina da Universidade de São Paulo Instituto do Coração São Paulo SP Brasil Instituto do Coração do Hospital das Clínicas da Faculdade de Medicina da Universidade de São Paulo, São Paulo, SP – Brasil

**Keywords:** Tabagismo, Análise da Randomização Mendeliana, Poluição do Ar, Parada Cardíaca, Saúde Pública

A parada cardiorrespiratória extra-hospitalar (PCREH) é uma manifestação grave de doença cardiovascular, com incidências anuais superiores a 350.000 casos nos Estados Unidos e na Europa.^1^ Apesar de décadas de melhorias nos protocolos de ressuscitação e da implantação de desfibriladores externos automáticos, as taxas de sobrevivência até a alta hospitalar normalmente permanecem estagnadas entre 5% e 15%. ^2-6^

Embora os médicos tradicionalmente se concentrem em fatores de risco metabólicos internos, como hipertensão e dislipidemia, a pesquisa apresentada no ABC Cardiol^7^ apresenta uma análise inovadora de fatores desencadeantes externos modificáveis – especificamente o tabagismo e a poluição do ar – utilizando a randomização mendeliana (RM) para ir além da simples associação e chegar a uma inferência mais robusta de causalidade.

Ainda assim, os achados justificam um otimismo cauteloso. Embora o estudo reforce a hipótese de que o tabagismo possa contribuir causalmente para a parada cardíaca, ele não fornece prova definitiva. Dada a heterogeneidade da PCREH e a complexidade das exposições relacionadas ao tabagismo, são necessárias mais evidências para esclarecer o papel deste último na morte súbita cardíaca (MSC) e seus mecanismos subjacentes.

## Indo além da associação – mas não além da incerteza

Uma inovação central deste estudo é o uso de RM para avaliar o papel causal tanto do tabagismo quanto da poluição do ar na parada cardíaca. As abordagens epidemiológicas convencionais frequentemente têm dificuldade em isolar o efeito de uma única exposição, pois múltiplos fatores de risco tendem a ocorrer simultaneamente. Por exemplo, viver em um ambiente poluído está frequentemente associado a um nível socioeconômico mais baixo, acesso limitado a serviços de saúde e condições de vida precárias. Da mesma forma, o tabagismo é comumente relacionado a outros comportamentos prejudiciais à saúde, incluindo alimentação inadequada e redução da atividade física.

A RM supera essas limitações utilizando polimorfismos de nucleotídeo único (SNPs) como variáveis instrumentais. Como as variantes genéticas são alocadas aleatoriamente na concepção e permanecem fixas ao longo da vida, elas são em grande parte independentes de fatores confundidores ambientais e comportamentais. Essa estrutura simula efetivamente um experimento aleatório natural, permitindo uma estimativa mais precisa dos efeitos da exposição ao longo da vida e fortalecendo a interpretação causal.

Contudo, a RM não elimina a incerteza. Mesmo quando bem conduzida, ela não consegue distinguir completamente os efeitos biológicos diretos de cadeias causais mais complexas, especialmente quando a exposição está inserida no acúmulo de risco cardiovascular ao longo da vida. Assim, os resultados apoiam a causalidade, mas não constituem prova definitiva de um efeito direto e específico do tabagismo na MSC.

## Robustez dos Resultados

Os autores implementaram diversas salvaguardas estatísticas para garantir a validade de suas descobertas. A robustez das variáveis instrumentais foi avaliada inicialmente usando a estatística F, que mede a força da associação entre os SNPs selecionados e a exposição. Valores acima de 10 são considerados suficientes para evitar viés de "instrumento fraco"; neste estudo, a estatística F média foi de 29,26, indicando instrumentos robustos.

A análise causal primária empregou o método de ponderação pela variância inversa, que agrega estimativas causais específicas de SNP em um único tamanho de efeito, dando maior peso às estimativas mais precisas. Essa abordagem identificou uma ligação causal estatisticamente significativa entre o tabagismo e a parada cardíaca (OR = 2,182, IC 95%: 1,14 - 4,19, p = 0,019).

Além disso, os autores aplicaram análises de sensibilidade adicionais para testar uma premissa fundamental da RM — que as variantes genéticas influenciam o desfecho apenas por meio da exposição de interesse. Para lidar com a pleiotropia horizontal (quando uma variante genética afeta o desfecho por meio de vias biológicas alternativas), eles utilizaram o MR-PRESSO, que detecta e corrige SNPs discrepantes que podem introduzir viés, e o MR-Egger, que identifica e ajusta a pleiotropia direcional, estimando se há um viés sistemático entre os instrumentos. Adicionalmente, uma análise de validação cruzada *Leave-One-Out* (LOO) confirmou que nenhum SNP isolado influenciou os resultados de forma desproporcional, reforçando a estabilidade das descobertas. Por fim, foi aplicada uma correção da taxa de falsos positivos para levar em conta os testes múltiplos.

## Sinal do tabagismo: importante, plausível, mas ainda não definitivo

Os resultados indicam que o tabagismo parece ter maior probabilidade de contribuir para a parada cardíaca do que simplesmente estar correlacionado a ela. O tabagismo tem sido associado a desequilíbrio autonômico, instabilidade elétrica ventricular, isquemia, disfunção endotelial, inflamação, trombogenicidade e remodelamento miocárdico, fatores que podem aumentar a suscetibilidade a arritmias malignas e parada cardíaca súbita.^8-11^ É importante destacar que os resultados corroboram a ideia de que parar de fumar pode reduzir substancialmente esse risco ao longo do tempo, reforçando seu papel como um fator modificável e clinicamente relevante.

## Poluição do ar: resultado nulo ou resolução limitada?

A ausência de uma associação causal significativa entre as métricas de poluição analisadas e a parada cardíaca deve ser interpretada com cautela. Um resultado nulo não equivale à evidência de ausência de efeito. O poder estatístico pode ter sido insuficiente para detectar efeitos pequenos ou moderados. A poluição do ar também é mais difícil de estudar por meio de RM do que o tabagismo, pois se trata de uma exposição externa espacial e temporalmente variável, e não de uma característica ou comportamento intrínseco. Além disso, seus efeitos cardiovasculares podem depender menos da exposição média ao longo da vida do que de picos de curto prazo, misturas de poluentes e interações com vulnerabilidade preexistente — dimensões que os modelos de RM atuais não capturam facilmente. Estudos observacionais, por outro lado, sugeriram repetidamente que aumentos agudos de material particulado podem precipitar PCREH em indivíduos suscetíveis.^6,12,13^

## Conclusão

Este estudo oferece uma contribuição importante ao fortalecer a hipótese de que o tabagismo desempenha um papel causal na parada cardíaca. A descoberta é biologicamente plausível e merece atenção. No entanto, cautela permanece essencial. A complexidade da PCREH, a interdependência das exposições ambientais e as limitações das abordagens atuais de RM significam que a causalidade ainda não pode ser considerada totalmente resolvida. Portanto, este trabalho representa um avanço na direção da compreensão do papel da exposição ao tabagismo na MSC, e não uma resposta definitiva, contribuindo para elucidar o papel da exposição ao tabagismo na MSC, ao mesmo tempo ressaltando-se a necessidade de validação adicional.
