# Supplementary material for: Evidências Genéticas Reforçam o Papel Causal do Tabagismo na Parada Cardíaca
Source: Arq Bras Cardiol. 2026 Apr 30;123(3):e20260238. [Article in Portuguese] doi: 10.36660/abc.20260238 (PMC13155793; doi:10.36660/abc.20260238)
Supplement: Translation

# Translation: Genetic Evidence Supports a Causal Role for Smoking in Cardiac Arrest

**DOI:** 10.36660/abc.20260238i

**Authors:** José S. L. Patané, Fernando R. Giugni

**Affiliations:** 1 São Paulo SP Brazil Instituto do Coração do Hospital das Clínicas da Faculdade de Medicina da Universidade de São Paulo, São Paulo, SP – Brazil

**Keywords:** Tobacco Use Disorder, Mendelian Randomization Analysis, Air Pollution, Heart Arrest, Public Health

Out-of-hospital cardiac arrest (OHCA) is a severe manifestation of cardiovascular disease, with annual incidences exceeding 350,000 cases in both the United States and Europe.^1^ Despite decades of improvements in resuscitation protocols and the deployment of automated external defibrillators, survival rates to hospital discharge typically stagnate between 5% and 15%.^2-6^

While clinicians traditionally focus on internal metabolic risk factors like hypertension and dyslipidemia, the research presented in ABC Cardiol^7^ provides a novel analysis of external modifiable triggers – specifically cigarette smoking and air pollution – utilizing Mendelian Randomization (MR) to move beyond simple association toward a more reliable causal inference.

Still, the findings warrant cautious optimism. While the study strengthens the hypothesis that smoking may causally contribute to cardiac arrest, it does not provide definitive proof. Given the heterogeneity of OHCA and the complexity of smoke-related exposures, further evidence is needed to clarify the role of smoking in sudden cardiac death (SCD) and its underlying mechanisms.

## Moving beyond association – but not beyond uncertainty

A central innovation of this study is its use of MR to evaluate the causal roles of both smoking and air pollution in cardiac arrest. Conventional epidemiological approaches often struggle to isolate the effect of a single exposure because multiple risk factors tend to co-occur. For instance, living in a polluted environment is frequently associated with lower socioeconomic status, limited healthcare access, and poorer living conditions. Similarly, smoking is commonly linked to other adverse lifestyle behaviors, including poor diet and reduced physical activity.

MR addresses these limitations by leveraging single-nucleotide polymorphisms (SNPs) as instrumental variables. Because genetic variants are randomly allocated at conception and remain fixed throughout life, they are largely independent of environmental and behavioral confounders. This framework effectively mimics a natural randomized experiment, enabling a cleaner estimation of lifelong exposure effects and strengthening causal interpretation.

Yet MR does not eliminate uncertainty. Even when well conducted, it cannot fully distinguish direct biological effects from more complex causal chains, especially when the exposure is embedded in lifelong cardiovascular risk accumulation. Thus, the findings support causality, but do not constitute definitive proof of a direct and specific smoking effect on SCD.

## Robustness of results

The authors implemented multiple statistical safeguards to ensure the validity of their findings. Instrument strength was first assessed using the F-statistic, which measures how strongly the selected SNPs are associated with the exposure. Values above 10 are considered sufficient to avoid weak instrument bias; in this study, the mean F-statistic was 29.26, indicating robust instruments.

The primary causal analysis employed the inverse-variance weighted method, which aggregates SNP-specific causal estimates into a single effect size, giving greater weight to more precise estimates. This approach identified a statistically significant causal link between smoking and cardiac arrest (OR = 2.182, 95% CI: 1.14 - 4.19, p = 0.019).

Furthermore, the authors applied additional sensitivity analyses to test a key assumption of MR - that the genetic variants influence the outcome only through the exposure of interest. To address horizontal pleiotropy (when a genetic variant affects the outcome through alternative biological pathways), they used MR-PRESSO, which detects and corrects for outlier SNPs that may introduce bias, and MR-Egger, which can identify and adjust for directional pleiotropy by estimating whether there is a systematic bias across instruments. Additionally, a Leave-One-Out (LOO) analysis confirmed that no single SNP disproportionately influenced the results, reinforcing the stability of the findings. Finally, false discovery rate correction was applied to account for multiple testing.

## Smoking signal: important, plausible, but not yet definitive

The results indicate that smoking appears more likely to contribute to cardiac arrest than to merely correlate with it. Smoking has been linked to autonomic imbalance, ventricular electrical instability, ischemia, endothelial dysfunction, inflammation, thrombogenicity, and myocardial remodeling, all of which may increase susceptibility to malignant arrhythmias and sudden arrest.^8-11^ Importantly, the findings support the notion that smoking cessation may substantially reduce this risk over time, reinforcing its role as a modifiable and clinically actionable factor.

## Air pollution: null result or limited resolution?

The absence of a significant causal association between the pollution metrics analyzed and cardiac arrest should be interpreted with restraint. A null result is not equivalent to evidence of no effect. Statistical power may have been insufficient to detect small or moderate effects. Air pollution is also harder to study through MR than smoking because it is a spatially and temporally variable external exposure, not an intrinsic trait or behavior. Moreover, its cardiovascular effects may depend less on lifelong average exposure than on short-term peaks, pollutant mixtures, and interactions with preexisting vulnerability—dimensions current MR models do not readily capture. Observational studies, by contrast, have repeatedly suggested that acute increases in particulate matter may precipitate OHCA in susceptible individuals.^6,12,13^

## Conclusion

This study provides an important contribution by strengthening the hypothesis that smoking plays a causal role in cardiac arrest. The finding is biologically plausible and worthy of attention. Nevertheless, caution remains essential. The complexity of OHCA, the interdependence of environmental exposures, and the limitations of current MR approaches mean that causality cannot yet be considered fully resolved. The work therefore represents an advance in direction rather than a final answer, bringing the field closer to understanding the role of smoke exposure in SCD while underscoring the need for further validation.
